# Diversified caregiver input to upgrade the Young Children’s Participation and Environment Measure for equitable pediatric re/habilitation practice

**DOI:** 10.1186/s41687-023-00627-2

**Published:** 2023-08-28

**Authors:** Vivian C. Villegas, Dianna L. Bosak, Zurisadai Salgado, Michelle Phoenix, Natalie Parde, Rachel Teplicky, Mary A. Khetani, High Value Early Intervention Research Group, L. Kuznicki, A. Pedrow, A. Howell

**Affiliations:** 1 Children’s Participation in Environment Research Lab, University of Illinois Chicago, Chicago, IL USA; 2 CanChild Centre for Childhood Disability Research, McMaster University, Hamilton, ON USA; 3 Department of Computer Science, University of Illinois Chicago, Chicago, IL USA; 4 Department of Occupational Therapy, University of Illinois Chicago, 1919 West Taylor Street, Room 316A, Chicago, IL 60612-7250 USA

**Keywords:** User-centered design, Caregivers, Environment, Diversity, equity, inclusion, Participation, Early childhood

## Abstract

**Background:**

Practitioner and family experiences of pediatric re/habilitation can be inequitable. The Young Children’s Participation and Environment Measure (YC-PEM) is an evidence-based and promising electronic patient-reported outcome measure that was designed with and for caregivers for research and practice. This study examined historically minoritized caregivers’ responses to revised YC-PEM content modifications and their perspectives on core intelligent virtual agent functionality needed to improve its reach for equitable service design.

**Methods:**

Caregivers were recruited during a routine early intervention (EI) service visit and met five inclusion criteria: (1) were 18 + years old; (2) identified as the parent or legal guardian of a child 0–3 years old enrolled in EI services for 3 + months; (3) read, wrote, and spoke English; (4) had Internet and telephone access; and (5) identified as a parent or legal guardian of a Black, non-Hispanic child or as publicly insured. Three rounds of semi-structured cognitive interviews (55–90 min each) used videoconferencing to gather caregiver feedback on their responses to select content modifications while completing YC-PEM, and their ideas for core intelligent virtual agent functionality. Interviews were transcribed verbatim, cross-checked for accuracy, and deductively and inductively content analyzed by multiple staff in three rounds.

**Results:**

Eight Black, non-Hispanic caregivers from a single urban EI catchment and with diverse income levels (*Mdn* = $15,001–20,000) were enrolled, with children (*M* = 21.2 months, *SD* = 7.73) enrolled in EI. Caregivers proposed three ways to improve comprehension (clarify item wording, remove or simplify terms, add item examples). Environmental item edits prompted caregivers to share how they relate and respond to experiences with interpersonal and institutional discrimination impacting participation. Caregivers characterized three core functions of a virtual agent to strengthen YC-PEM navigation (read question aloud, visual and verbal prompts, more examples and/or definitions).

**Conclusions:**

Results indicate four ways that YC-PEM content will be modified to strengthen how providers screen for unmet participation needs and determinants to design pediatric re/habilitation services that are responsive to family priorities. Results also motivate the need for user-centered design of an intelligent virtual agent to strengthen user navigation, prior to undertaking a community-based pragmatic trial of its implementation for equitable practice.

**Supplementary Information:**

The online version contains supplementary material available at 10.1186/s41687-023-00627-2.

## Background

Practitioner and family experiences of pediatric re/habilitation can be inequitable, thereby creating a barrier to achieving the quadruple aim of services which includes optimizing patient and provider service experiences [1, 2]. An early opportunity to promote Justice, Equity, Diversity, and Inclusion (JEDI) in a pediatric re/habilitation service context and workflow is presented when designing an early intervention (EI) service plan. EI programs typically serve families with children 0–3 years old in the U.S. and aim to improve child and family outcomes (e.g., participation in valued activities, skill development) by providing services in the child’s natural environment [3, 4]. JEDI principles can be baked into assessments that practitioners use and make available to EI families. These tools guide shared decision-making and service planning with children and families. For example, a video introducing an assessment and/or its items with diverse imagery of children participating in activity is a subversive way to ensure that families can relate to questions they are asked and share their priorities for intervention [5]. Assessments therefore should be designed to foster habits for equitable and inclusive decision-making for service design and improvement.

Participation and Environment Measure (PEM) is a two-part electronic approach to reinforce data-driven, collaborative, and agile decision-making for pediatric re/habilitation service design and improvement. For families of young children, the first part of PEM is the Young Children’s Participation and Environment Measure (YC-PEM), an evidence-based and promising electronic patient-reported outcome (e-PRO) assessment [6–8] that gives caregivers a valid, reliable, and feasible way to describe their young child’s current and desired participation, evaluate environmental impact on participation, and describe strategies they have tried to support participation in those activities where change is desired. The YC-PEM assessment is a promising e-PRO because it may also detect change in participation over time [9, 10]. Participation and Environment Measure-Plus (PEM+), the second part, is a feasible, acceptable, and promising care planning tool that guides caregivers online to build on their YC-PEM responses to prioritize unmet needs, set specific goals, and exchange participation-related strategies for goal attainment [11–14].

Khetani and colleagues pursued concept mapping, secondary data analyses, and/or qualitative research with caregivers of young children with developmental need (including historically minoritized caregivers) to develop and validate original YC-PEM content and layout [15–18] and PEM+ design [12–14, 19]. They envisioned its need to be useful in accelerating family-centered service design with individual families and as a common data element in health services research for quality service improvement [20]. Construct validation continues for its original version [7, 21] and culturally adapted versions [22]. There has also been effort to examine common adaptation needs, critically appraise and confront issues of JEDI in its design and functionality to support user navigation (e.g., border color, activity illustrations; [5, 23–25]), and better recruit, retain, and describe more diverse users [26, 27]. During these efforts to further validate and culturally adapt the original YC-PEM assessment, there was increasing recognition by caregivers, trainees, providers, and researchers that it could still benefit from further content and navigation upgrades to optimize its implementation for equitable pediatric re/habilitation research and practice. In response, Khetani and colleagues developed and solicited for feedback on select content upgrades that may ensure that its phrasing fully reinforces a consistent focus on participation and adequately captures the full range of environmental factors that can support or hinder participation [23, 28–31]. Specifically, Khetani and colleagues hypothesized that providing families with opportunity to disclose the impact of racism and other forms of discrimination on their young child’s participation in activities could improve the application of JEDI principles to this assessment by centering this aspect of their lived experience while navigating their home, daycare/preschool, and community environments. Navigation upgrades may ensure that YC-PEM supports shared decision-making for families, similar to benefits observed with conversational agents designed to support minoritized patients in healthcare [32]. For example, racially minoritized patients may perceive virtual agents to be less judgmental and more available than providers [33, 34].

PEM content and navigation upgrades may help rehabilitation practitioners build habits for adequately reaching and guiding diverse caregivers through assessment of their unmet needs to design participation-focused pediatric re/habilitation services. Content upgrades may also afford for more robust analyses of environmental impact on participation for groups of families with known disparities [35, 36] in accessing pediatric re/habilitation services. End-user feedback is important to ensuring its clarity and value prior to deployment.

The purpose of this study is therefore two-fold: (1) to examine caregiver perspectives of the clarity and relevance of proposed YC-PEM content modifications, in two ways: (1a) content modifications to reinforce participation-focused assessment; and (1b) content modifications to screen for caregiver perspectives of racism and other forms of discrimination when appraising environmental impact on their young children’s participation; and (2) to examine caregiver experience with dialogue agents to define functional requirements needed to pursue user-centered design of an intelligent virtual agent for supporting PEM user navigation.

## Methods

Cultural adaptation methods were followed [37] throughout the project to guide sampling, data collection, and analysis methods as described below. Ethical approval was obtained by the Institutional Review Board at the University of Illinois (protocol #2020-0908).

### Participants

Caregivers were recruited by EI staff through convenience sampling and during a routine service visit. The sample size estimate was based on prior work involving cognitive testing of original and culturally adapted PEM versions [22, 23] according to best practice guidelines [37] (M. Khetani, personal communication, June 12, 2023). For this study, a relatively smaller sample size was projected for gathering caregiver feedback because we were seeking to finalize select content upgrades for an established assessment. Caregivers were eligible to participate if they: (1) were 18 + years old; (2) identified as the parent or legal guardian of a child 0–3 years old enrolled in EI services for 3+ months; (3) read, wrote, and spoke English; (4) had Internet and telephone access; and (5) identified as a parent or legal guardian of a Black, non-Hispanic child (BNH) or as publicly insured.

The fifth inclusion criterion is based on known social disparities in pediatric re/habilitation access and use among racially and ethnically diverse families (e.g., BNH children enrolled in EI have 75% lower odds of receiving physical therapy services and receive one hour less of EI services per month, as compared to White, non-Hispanic peers) and socially disadvantaged families (e.g., publicly insured children had less intensive EI therapies; [38, 39]). This inclusion criterion also responds to historically low representation of BNH families (0.8–6.5%) and socially disadvantaged families (10.4–20.9%) in prior YC-PEM development, validation, and health services and implementation research inclusive of EI families [8, 28, 30, 31, 40].

Service coordinators in early childhood research groups, including those at the study site, co-designed recruitment materials. Eight eligible and interested caregivers were directed via flyer weblink to a project website, to create a user account and route into REDCap to enroll [41, 42]. To support participant retention, research staff: (1) initiated 1–5 personalized email(s) and up to 12 text message reminder(s) for scheduled caregivers, (2) gave caregivers choice to opt in/out of receiving a $40.00 electronic gift card; and (3) offered caregivers immediate access to their online YC-PEM summary report(s) to download, store, and share as a PDF with their child’s service team.

### Data collection

Data collection was completed in three rounds (2–4 caregivers per round), per best practice standards for using cognitive interviews to culturally adapt pediatric participation assessments [37, 43, 44]. For each round, caregivers entered REDCap to confirm study eligibility, used text and/or video options to provide informed consent and HIPAA authorization, and completed a sociodemographic questionnaire. All participating caregivers opted for a semi-structured videoconferencing session (55–90 min each) with research staff, whereby caregivers screenshared while completing the YC-PEM assessment and shared their feedback based on how they understood reworded items and their reactions to proposed content modifications. Specifically, 1–2 research staff (VV, ZS) used an interview guide to prompt for caregiver feedback when they encountered a YC-PEM assessment item with proposed content modifications (see Additional file 1: Appendix). Seeking participant feedback shortly after completing a question allows them to offer their thoughts when presumably fresh [45–47]. Following each round, the interview guide was modified to include additional prompts based on caregiver feedback to proposed content modifications until data saturation was reached.

After providing item-level feedback on content modifications, caregivers shared their experience with virtual agents in their everyday life to define core intelligent virtual agent functionality that can support user navigation through the PEM option. Research staff asked caregivers open-ended and closed-ended questions at the end of the interview such as familiarity and interaction frequency with virtual agents, tech-related skills, and important features to include in the design of a virtual agent (see Additional file 1: Appendix).

### Data analysis

Videoconferencing sessions were screen and audio-recorded. Prior to main analyses, research staff (VV, ZS, DB) created a transcript for each participant with their narrative responses from auto-transcriptions. Transcript content was cross-checked for accuracy, imported into NVivo 13.0, and sorted to the corresponding interview guide question prior to main analyses.

Data analysis took place in three rounds (2–4 interviews per round). For Aim 1a, frequency counts were applied to closed-ended items to identify the extent of user agreement with each proposed content modification [48]. For Aim 1b and Aim 2, inductive content coding was completed iteratively. Two to three team members (VV, ZS, DB) independently content analyzed open-ended responses for each written probe in the interview guide, to identify recurrent ways that users understood reworded items, reacted to proposed modifications, suggested further modifications to assessment items, and related to virtual agents [49]. Coders discussed discrepancies to reach consensus and further refine the codebook [14, 23]. Select preliminary coded content were reviewed by key informants (MK, VK, NP, MV, SF) to ensure trustworthiness of categories (e.g., coded content fit the category, categories were distinct, and category labels used language from coded text), resulting in some categories being further collapsed.

During data collection and analysis, self-reflexivity was used to ensure authenticity and trustworthiness of findings, by acknowledging researchers’ intersectional identities (e.g., Black, Indigenous, and people of color (BIPOC) identifying) and prior experiences (e.g., training in rehabilitation professions, belonging to anti-racist research labs) [49–52]. Authors also have prior experience with content coding caregiver data about their child’s participation in services and activities and are therefore familiar with caregiver perspectives of their child’s participation [21, 53]. These professional and lived experiences shaped decisions about content modifications and approaches to ensuring interpersonal safety during data collection.

## Results

### Caregiver characteristics

As shown in Table 1, eight Black, non-Hispanic caregivers from a single urban EI program located in the Mountain U.S. region enrolled in this study. Most caregivers identified as the mother or female legal guardian (87.5%) of their child receiving EI services. Many caregivers reported having earned a high school degree or equivalent (50%) and with family income below the U.S. median (87.5%) [54]. Only two caregivers reported full-time employment (25%).

**Table 1:** Child and caregiver characteristics (N = 8)

Characteristic	n (%)
Caregiver gender identity	
Mother or female guardian	7 (87.5)
Non-binary	1 (12.5)
Caregiver race and ethnicity	
Black or African American	8 (100)
Not Hispanic, Latinx, or Spanish Origin	8 (100)
Caregiver education level	
High School graduate, diploma or equivalent	4 (50)
Graduated college/university	1 (12.5)
Some graduate coursework	2 (25)
Graduate degree	1 (12.5)
Caregiver employment	
Not currently working	3 (37.5)
Part-time	3 (37.5)
Full-time	2 (25)
Family income, $*	
Less than 5000	3 (37.5)
15,001–20,000	2 (25)
30,001–40,000	1 (12.5)
90,001–100,000	1 (12.5)
Child age (mo)	
0–12	1 (12.5)
12–24	3 (37.5)
24–36	4 (50)
Child gender identity	
Male	4 (50)
Female	4 (50)

*Data not reported by one participant

### Caregiver feedback guiding YC-PEM content upgrades (Aim 1)

All eight caregivers shared feedback on the clarity and value of modifying the content of one YC-PEM participation assessment scale and five of its environmental items (see Additional file 1: Appendix).

#### Caregiver feedback on YC-PEM participation assessment

Three of eight caregivers described their effort when encountering the newly phrased response options for reporting on type(s) of participation change desired, by showing how they arrived at their response(s). As one caregiver shared,

it's grouping everything in one. Like getting dressed, it’s grouped so I wouldn't because maybe she's lacking in one area and she's not lacking in another area. So I did wanna put maybe ‘yes, help more’, but then I feel like she helps. I feel like she helps enough with getting dressed and brushing her teeth. Yeah. So that's the reason why I was teetering between those 2 answers [Caregiver (CG) 1].

Similarly, another caregiver shared their effort for deciding how to quantify interactions for a broader type of activity and struggled to decide on relevant response options.

#### Caregiver feedback on YC-PEM environmental assessment

Caregivers provided four types of feedback on proposed content modifications to environmental assessment item examples, including three points of feedback to improve comprehension (clarify item wording, remove or simplify terms, and add item examples), and feedback about how they understand environmental content modifications to screen for experiences of interpersonal and institutional discrimination as they impact opportunities for participation (see Table 2).

**Table 2:** Caregiver feedback on YC-PEM content modifications

Proposed modification	Rationale	Codes			
**Participation assessment**					
*Yes, be more interactive* rephrased as *Yes, interact more*	Upgrades to participation assessment further clarifies that changing participation is not about changing the child to ‘be more’; rather, changing participation is about creating opportunities for the child to get more involved in the type of activity	No, was not stuck Yes, was stuck			
*Yes, be more helpful* rephrased as *Yes, help more*					

Environmental assessment					
Proposed modification	Rationale	Codes			
		Clarify items	Remove or simplify terms	Add item examples	Understanding environmental content modifications
Home The attitudes and actions of babysitters, therapists, and other professionals who care for your child at home *(e.g., microaggressive/* *allyship behaviors)* Community The attitudes and actions of other members of the community towards your child (e.g., *microaggressive/allyship behavior of* staff at stores and restaurants, instructors, coaches, childcare provider, other families)	Upgrades to environmental assessment include anti-racist terms (e.g., microaggressions, implicit bias) in the item examples, caregivers may be inclined to consider, report, and share their expertise about issues of this type	Home Lack of comprehension Community Confusion with location and job roles	Home Anti-racist terms may not be needed Assumed education level Include full question Community Anti-racist terms may be unnecessary or triggering Learning opportunity Use of simple words	Home Allyship example Microaggression example More descriptive example Community Microaggression example	Home All services are virtual Anti-racist term description Child’s interactions with individuals Monitoring who comes in the house Community Caregiver judged based on child’s behavior No one that looks like the child Positive community interactions support participation Strangers in the community
Community Your child’s relationships with peers *(e.g., neighbor)*		N/A	Community Neighbor may not be needed	Community Expand or specific examples to clarify neighbor	Community Building social skills Difference in peer intimacy levels
Home Policies *(e.g., inclusive/discriminatory policies pertaining to a residence or workplace, such as family leave or working from home, time off, work hours)* Community Policies *(e.g., inclusive/discriminatory policies by neighborhood, childcare, or employer)*		Home Lack of comprehension Community Confusion over neighborhood policy Understands but can’t explain	Home Assumed health literacy Parenting policies Community Referring back to previous policy questions	Home More descriptive examples Community Specific neighborhood examples	Home Residence policies Work flexibility and acknowledgement of caregiver priorities Community Challenging childcare policies and procedures Safe and inclusive communities Securing and sustaining employment

##### Clarify items

Seven of eight caregivers expressed the need to clarify the point of reference for items with proposed content modifications in the home environment section. One caregiver shared uncertainty about the anti-racist term being applied to the child or their environment and shared:

I think you should put positive or negative behaviors when a professional who cares for your child comes into your home cause I’m not gonna lie I didn’t know what the microaggressive – I just saw aggressive and behavior. I was like, okay, it’s gotta be with the kid but it wasn’t (CG4).

Similarly, another caregiver expressed they were unsure how policies being asked about pertained to the child, sharing that for appraising the impact of employer policies on participation, “I was immediately confused 'cause I thought … he's not working, I am. So I was like confused” (CG7).

Six of eight caregivers sought similar clarity when encountering similar proposed content modifications in the YC-PEM community environment section. One caregiver recommended regrouping types of community members for clarity about the point of reference, because “working at a restaurant or a store … Children are people, they are going to come in. But instructors and coaches and childcare providers … that's part of your job description” (CG1). Caregivers expressed uncertainty when asked to appraise the impact of their neighborhood policies on participation by sharing, “Honestly, this is one of those questions … that, like kind of threw me off a little bit more. I don't have anything off the top of my head” (CG3).

##### Remove or simplify terms

Seven of eight caregivers suggested that new anti-racist terms be removed or simplified to increase caregivers’ comprehension of modified items.

Some caregivers suggested removing anti-racist terms in the home environment section because “I don't necessarily think that at his age microaggressions are something that he is going to be affected by right now” (CG7). For community, one caregiver suggested removal since “whenever we use those words, they're kind of like trigger words for other people and they start to automatically think about the negatives of that” (CG3).

Alternatively, some caregivers proposed simplifying the terms used. For home environment, one caregiver suggested simplification because “not every parent has the privilege of being in a higher education or getting the privilege of knowing in some ways saying they are synonyms. So it's better to use a very simple word that everybody can understand it” (CG 5). For community, caregivers proposed “using easier words” (CG4) to assist in item comprehension, stating that “Just a simple, negative or positive behaviors … I think simple words to make it clear what you're looking for” (CG3).

##### Add item examples

Seven of eight caregivers requested more descriptive item examples. For home, one caregiver proposed more description of the examples and shared, “I just feel like the other examples that you guys have in parentheses are like, pretty much I would say easy for everyone to like know the definition or understand what that means. Maybe a little bit more descriptive with the example?” (CG1). Another caregiver shared personal examples of anti-racist terms, such as illustrating how “We know that that person (*babysitter*) is an ally, not just to his development, but to help us as well” (CG3).

Similarly, seven of the eight caregivers requested additional item examples for community items, such as by adding “friends and family” (CG 7) and “child to child relationship” (CG3) when describing relationships, and adding “popping fireworks at like 1, 2 in the morning” (CG1) to illustrate policies that can impact community participation. Multiple caregivers shared their experiences with what we classify as microaggressions, to describe what it is like to attempt to participate in play at their local parks, where “kids are so mean and parents are not saying anything to their mean kids” (CG5) or children saying “he can't play over here” or “we own this” (CG7).

##### Understanding environmental content modifications

All eight caregivers were prompted by the YC-PEM proposed content modifications to describe how they relate to and respond to experiences with interpersonal and institutional barriers to participation, including experiences with racism, at home and/or in the community.

At home, one caregiver defined microaggression as “politely aggressive” and allyship as “friendship behavior” (CG5). As a response, multiple caregivers expressed they monitor who comes into their home environment stating “I don't do bad energy. You ain’t even gonna step in my house” (CG4) or that they are “Trying to do a very good job of not having that (*microaggression*) in the house” (CG3). Institutional policies that impacted their home environment included flexible residential policies, such as having “a very easy going HOA for the most part” (CG3), and flexible workplace policies, such as “I mean if I can't get off of work, then they'll have to go somewhere else” (CG2). One caregiver stated:

I normally don't get jobs that require full time because I do have kids under the age of 3. I'm also pregnant, so I kind of get jobs that work with my schedule and I’ll let them know … [that it] works for me. Because if it doesn't work for me, I'm not gonna work there (CG4).

In the community, caregivers again shared how they relate to and respond to interpersonal and institutional barriers, including those involving racism. Interpersonally, caregivers expressed the importance of positive social interactions because “when he's around peers… it just helps him build social skills” (CG2) and “the only environments we're around are usually very helpful. There are people who are encouraging, there's other babies that are around his age” (CG7). Institutional practices and policies were described as impacting caregiver access to safe and inclusive communities. As one caregiver shared:

I mean, where we live, she goes and does activities but their attitudes are still OK. I mean, when we go do activities like her music and me class, I mean there’s no one that looks like her, but there’s still positive attitudes towards her (CG6).

However, caregivers then struggle with challenging childcare policies, such as “If you're late, it's $5 a minute” (CG1) that restrict their access to opportunities for community participation.

### Caregiver feedback to improve user navigation (Aim 2)

When caregivers interact with virtual agents in their everyday life (e.g., Siri, Alexa), they value professional tone (n = 6) and shared struggles with voice recognition (n = 5). In the absence of a virtual agent for YC-PEM completion, caregivers asked the interviewer for help (n = 4), reread the question (n = 3), or skipped the question (n = 2). Caregivers also offered three core functions for a virtual agent to professionally support their navigation.

#### Read question aloud

Five caregivers requested the virtual agent to read YC-PEM items aloud. Caregivers reported, “Something that we as parents can click on and read or even have like a[n] audio where it reads to you 'cause some people have a hard time reading on their own versus somebody reading it out loud to them” (CG8) and “If it can read the question for me because I'm lazy, but even sometimes I think just understand it 'cause I read some of them several times” (CG6).

#### Visual and verbal prompts

Two caregivers also shared ideas for how a virtual assistant could prompt for and facilitate their responses, by (1) having more video features, since “I like how you guys did the video first. So I would say the video feature first. Cause it gave me an idea of what the questions was going to be about” (CG4) and (2) including prompts to choose the best answer, to “just like maybe say, choose the answer that suits you best. Giving information on how to complete the survey so that you guys get the best results” (CG2).

#### More examples and/or definitions

**Fig. 1:**
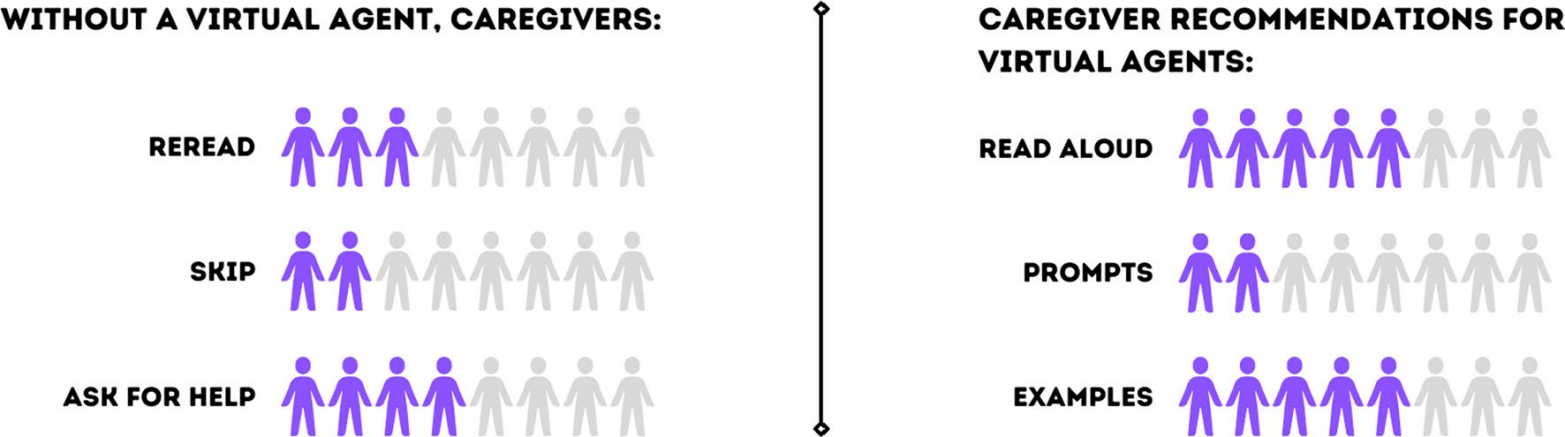
Caregiver Feedback to Improve YC-PEM User Navigation

Five caregivers shared that a virtual agent could provide more examples [e.g., “You know how you gave me examples if I was stuck on a question? … Explain to me how that fits within that question. So, just being able to I guess understand the questions… Yeah, a different example” (CG1)] and/or definitions [e.g., “I think provided definitions for some of those larger words will be useful” (CG8)] for YC-PEM assessment completion (Fig. 1).

## Discussion

Pediatric re/habilitation espouses the importance of equitably partnering with families to design meaningful service plans to improve their child’s participation in valued activities. This study leverages the expertise of historically minoritized caregivers to confirm and extend knowledge about ways to improve user comprehension and navigation of an evidence-based assessment of participation.

*Conceptualizing Involvement as Transactive and Interdependent.* Caregivers in this study repeatedly confirmed that they expended effort but could arrive at their responses when provided with different phrasing for response options to describe the type(s) of participation change they desire. This revised phrasing of response options makes more explicit that the participation concept denotes an act rather than a trait of the child, an approach that is congruent with the contemporary family of Participation-Related Concepts (fPRC) framework that employs verbs (e.g., choosing, complying) to denote the transactive relationship between a child’s participation and its related concepts that are often intrinsic to the child (e.g., their preferences) [55, 56].

Caregivers did not propose new response options, further confirming that the original verbs of ‘interact’ and ‘help’ remain robust indicators of the involvement dimension for the participation concept [16]. This finding reinforces the conceptual distinction between participation and its related concepts, such as intrinsic factors like child interest. As YC-PEM has been primarily shaped by the perspectives of BIPOC identifying caregivers situated in the U.S., the coexistence of individualistic and collectivist parent belief systems may have shaped their expectations for interdependence when describing their young child’s involvement in activities [57, 58]. This focus on interdependence distinguishes participation from its intrinsic concepts (i.e., activity competence, preferences, sense of self) and differentiates it from more established approaches to measuring participation [59, 60].

*Strengthening How Caregivers Relate and Respond to Environmental Content.* Caregivers in this study proposed ways to clarify, remove/simplify, or add examples to ensure their comprehension, and they repeatedly shared how they could relate and respond when introduced to select anti-racist terms for evaluating the impact of a discriminatory home or community environment. Results suggest that caregivers can be prompted to comprehend and consider their experiences of inequity that could be grounded in experiences of racism, both interpersonally and institutionally [2, 61]. Following YC-PEM content modifications, there is need for subsequent studies to systematically apply these YC-PEM data into the complex clinical task of goal setting, if the data are to consistently demonstrate provider commitment to screening for its impact on participation. To best include these data in the goal setting process, EI practitioners proposed that the YC-PEM be administered prior to a child’s upcoming evaluation of progress, and that both practitioners and families access summary reports [30]. In related work, EI caregivers and providers have piloted a program-specific decision-support tool for integrating YC-PEM results to guide service plan development [40].

*Agent Support for Equitable User Navigation through Assessment Approach.* Artificial intelligence (AI) is novel in participation-focused goal setting applications [59, 62], but our results clearly indicate that caregivers anticipate benefits to its use in this context. This is unsurprising, given prior studies involving conversational agents in other healthcare contexts focused on supporting minoritized patients [32–34].

Caregivers emphasized several distinct desires for agent functionality. First, caregivers wanted the agent to recognize and relate to them in a professional manner. Research has shown that racially and ethnically diverse and socially disadvantaged families are more likely to engage with verbally dominant and less patient-centric clinicians [63]. We speculate that this may shape their wishes for the agent’s behavior; along these lines, prior studies have shown racially minoritized patients may perceive conversational agents to be less judgmental and more available [33, 34]. Caregivers also emphasized the value of accessing support to read questions aloud, prompt for and facilitate responses, and offer examples. Literature supports this need, showing that frequency of repeat requests and user questions are predictive of engagement in virtual agent-based healthcare interventions [64]. Similarly, we successfully piloted automated prompts to reinforce the scale points selected (e.g., to confirm user response to first involvement item in each YC-PEM section) [30], and can also link the user back to the portion of the YC-PEM introductory video containing sample caregiver strategies. The potential for automated classification of these caregiver strategies could also offer agent functionality for supplying users with relevant examples of strategies during YC-PEM completion [65, 66].

Future work may benefit from expanding our research group to include those study participants who have expressed interest to serve as patient advisors during agent testing, using these three points of feedback to frame user evaluation of a prototypic intelligent agent.

### Limitations

Study results should be interpreted in light of several limitations. Our fifth inclusion criterion limited our access to Black, Hispanic families who may share discriminatory experiences when trying to support their young child’s participation in valued activities [50]. We plan for broader inclusion criteria specific to caregiver race/ethnicity in planned pragmatic trials of YC-PEM implementation in EI. Similarly, we struggled to engage a local therapeutic preschool program for recruiting caregivers that met these inclusion criteria, thereby limiting our reach to families of children 3–5 years old and in a different geographic region. Lessons learned can strengthen development of this research infrastructure in planned multi-site pragmatic trials that include the upgraded YC-PEM paired either with its companion decision-support tool [27] or PEM + care planning application [19, 26]. In addition, results could be specific to perspectives gathered during COVID-19 that could have altered caregiver expectations and opportunities for participation and increased their familiarity with using technologies for telehealth rehabilitation services [67, 68] and everyday life [69]. Finally, recruitment, retention, and data collection of historically minoritized EI families took longer than anticipated, signaling the need to consider more efficient analytic techniques like RADaR [70] when examining stakeholder perspectives of supports, barriers, and strategies for PEM implementation during a planned pragmatic trial.

## Conclusion

Family-centered care in EI requires an understanding of the unique positioning of each child and family in their environments, such that service providers can collaborate with families to tailor their approach, activities, and recommendations to support full participation in a child’s natural environment. Study results report on how the YC-PEM assessment was upgraded with caregiver input to finalize its content for participation assessment and screening for experiences of racism and other forms of discrimination, and to examine experiences with a virtual agent to define relevant core functionality. Caregivers gave concrete suggestions to simplify the language used for anti-racist items, modify questions by breaking down its component parts, and provide more definitions/examples. Environmental and institutional barriers to participation were elicited using the tool. The virtual agent could be utilized to support use of the tool by reading questions aloud, providing visual/verbal prompts, and more definitions/examples. Content modifications will improve the validity of the tool in capturing systemic inequalities that can impact children’s participation in a variety of environments. Development of a prototypic virtual agent is underway to support navigation for caregivers and may increase YC-PEM use for more personalized goal setting and service planning.

### Supplementary Information

**Additional file 1**. Appendix.

## Data Availability

Not applicable.

## Abbreviations

AI: Artificial intelligence
BIPOC: Black, Indigenous, and people of color
BNH: Black, non-Hispanic
CG: Caregiver
EI: Early intervention
e-PRO: Electronic patient-reported outcome
fPRC: Family of participation-related concepts
JEDI: Justice, equity, diversity, and inclusion
PEM: Participation and Environment Measure
PEM+: Participation and Environment Measure Plus
YC-PEM: Young Children’s Participation and Environment Measure
