# Supplementary material for: Diversified caregiver input to upgrade the Young Children’s Participation and Environment Measure for equitable pediatric re/habilitation practice
Source: J Patient Rep Outcomes. 2023 Aug 28;7:87. doi: 10.1186/s41687-023-00627-2 (PMC10462549; doi:10.1186/s41687-023-00627-2)
Supplement: Supplementary file 1 — Additional file 1. Appendix. [file 41687_2023_627_MOESM1_ESM.docx]

**Appendix**

*Cognitive Interview Guide*

**A. Interviewer Introduction:**

“Hello! My name is XXX and this is XXX. We work on this research project at UIC in Chicago. Thank you for taking the survey and agreeing to talk with us. This survey was developed with family input about a decade ago and we have recently made some changes. We want to make sure it is still easy to understand and fill out. So today we are coming back to families like yours to get your input.

I actually didn’t write the survey questions, so don’t worry about hurting my feelings with your comments about them as we talk. My job is to find out what’s wrong with them.

Do you have any questions for us? Let’s begin and start the recording.”

**B. INTRODUCTION QUESTIONS:**

1. Tell us about your child (age, EI/ECSE experience, diagnoses, etc.)
2. What is your occupation?
3. What is your favorite activity to do with your child?

**C. THINK ALOUD WARM-UP QUESTION:**

“Today we will be using a method called “thinking aloud”. Thinking aloud may be new and unfamiliar to you, but please know there are no wrong answers. I am only interested in knowing what is going through your mind. Any information you provide during this pre-interview will not be used in the project; it is merely to help you become familiar and comfortable with the ‘think aloud’ process. Now I’d like to ask you a ‘warm-up’ question to introduce you to the think aloud process.”

1. Try to visualize the place where you live, and think about how many windows there are in that place. As you count the windows, tell me what you are seeing and thinking about. (Willis, 1994)

“We will now begin taking the survey. At some points we will have you finish a section and then we will talk about it. At other times, we will ask you questions about every other item. Thank you in advance for your flexibility! Let’s begin.”

**D. YC-PEM WARM-UP QUESTIONS:**

“For this first section, Basic Care Routines, you will complete all four activities and then we will ask you to choose one to talk more about. Take your time and let me know when you are done and ready to talk.”

Basic Care Routines: “What activity do you want to talk about first?”

*Caregiver selects one activity (i.e., getting rest, personal care management, getting clean, mealtime)*

1. How do you understand what is meant by these example activities?

*Alternative Ways:*

*Was the description of this activity area clear?*

*In other words, did you know what we meant by the activity area?*

1. Were there any words that you think we could make easier?

*Probes:*

*If yes, which words?*

*What do you think is a better word?*

1. We made some changes to Question C.

Describe to me how you arrived at your response in Question C? And how sure are you about your answer?

*Probes:*

*Were you ever stuck between two options?*

*Why did you choose or not choose option XXX?*

**E. ENVIRONMENT QUESTIONS:**

“For this next section, Home Environment, you can choose the first item we talk about and then we will ask you about a few specific items. Feel free to ask us to repeat a question or if you need more time.”

Environment: “Out of numbers 1-8, what environmental item do you want to talk about first?”

*Caregiver selects one home environmental factor (i.e., physical layout, sensory qualities of home, physical demands, cognitive demands, social demands, child’s relationships with family members, attitudes/actions of others at home, policies)*

1. How do you understand what is meant by these different factors in the environment?

*Alternative Ways:*

*Was the description of this environmental factor clear?*

*In other words, did you know what we meant by the environmental factor?*

1. Were there any words that you think we could make easier?

*Probes:*

*If yes, which words?*

*What do you think is a better word?*

1. Describe how you got to your answer for this question.

*Probes:*

*Were you ever stuck between two options?*

*Why did you choose or not choose option XXX?*

**F. ENVIRONMENT (HOME) CONTENT QUESTIONS:**

*if caregiver selects item 7 or 8 for their personal choice, then ask about item 6 in addition to the unselected item 7/8 (i.e., caregiver personally selects item 7, then ask about items 6 and 8)

Home Environment: 7 (Attitudes/Actions) and 8 (Policies)

1. How do you understand what is meant by these different factors in the environment?

*Alternative Ways:*

*Was the description of this environmental factor clear?*

*In other words, did you know what we meant by the environmental factor?*

1. Were there any words that you think we could make easier?

*Probes:*

*If yes, which words?*

*What do you think is a better word?*

1. Describe how you got to your answer for this question.

*Probes:*

*Were you ever stuck between two options?*

*Why did you choose or not choose option XXX?*

1. Are there any other questions on this page that you think would benefit from new examples?

*Probes:*

*If so, what words would you use?*

*Are there any other examples that we missed?*

**G. ENVIRONMENT (COMMUNITY) CONTENT QUESTIONS:**

“For this next section, Community Environment, we will ask you about a few specific items. Feel free to ask us to repeat a question or if you need more time.”

Community Environment: 6 (Attitudes/Actions), 7 (Child’s Relationships with Peer), and 10 (Policies)

1. How do you understand what is meant by these different factors in the environment?

*Alternative Ways:*

*Was the description of this environmental factor clear?*

*In other words, did you know what we meant by the environmental factor?*

1. Were there any words that you think we could make easier?

*Probes:*

*If yes, which words?*

*What do you think is a better word?*

1. Describe how you got to your answer for this question.

*Probes:*

*Were you ever stuck between two options?*

*Why did you choose or not choose option XXX?*

1. Are there any other questions on this page that you think would benefit from new examples?

*Probes:*

*If so, what words would you use?*

*Are there any other examples that we missed?*

**H. VIRTUAL AGENT DESIGN QUESTIONS:**

“We have now completed the YC-PEM. For this next section, we will ask you about your YC-PEM experience and how you interact with technology. Feel free to ask us to repeat a question or if you need more time.”

1. How familiar are you with YC-PEM?
2. Have you completed YC-PEM previously?

*Probes:*

*If so, how many times (0, 1-2, or more than 3)?*

*If so, have the results been used in your service planning meetings?*

1. Do you use bots or virtual agents for your day-to-day tasks?

*Probes:*

*If so, what are some virtual agents with whom you commonly interact?*

*Examples: Siri, Alexa, Cortana, Google Assistant, or specific bots on shopping/e-commerce websites*

*If so, what is your frequency of use? (Adapted from the paper here:* [*https://dl.acm.org/doi/pdf/10.1145/3411764.3445445*](https://dl.acm.org/doi/pdf/10.1145/3411764.3445445)*)*

1. To what extent would you consider yourself to be tech savvy?
2. What features are important to your experience when interacting with a virtual agent?

*Probes:*

*Examples: Multimodal input/content delivery, system-driven dialogue, or user-driven dialogue*

1. With which platforms do you feel comfortable?

*Probes:*

*Examples: Facebook Messenger, Slack, Email, or SMS*

1. What are the main things with which you’d like a virtual agent to be able to help when completing YC-PEM?
2. What kind of information do you think would be useful for a virtual agent to provide when completing YC-PEM?
3. How would you expect a virtual agent that is assisting you with YC-PEM to act?

*Probes:*

*Examples: Professional, casual, formal, or informal*

1. When you are confused about something when completing YC-PEM right now, what do you do to resolve your confusion?

**I. WRAP-UP QUESTIONS:**

“Thank you for taking the time to talk with us today. As we wrap up, we have just a few final questions to ask.”

1. Is there anything else you want to share with us?

“Thank you, that concludes our questions. Within the next week you will receive an electronic gift card in your email if you opted for payment. Please feel free to email us with any further questions. Goodbye!”
